# Determinants of long act reversible contraceptive utilization among HIV positive reproductive age women attending ART clinic in South West Ethiopia

**DOI:** 10.1186/s40834-023-00227-x

**Published:** 2023-05-04

**Authors:** Bilisumamulifna Tefera Kefeni, Sitota Tesfaye, Kenbon Bayisa, Ebissa Negara, Feyiso Bati

**Affiliations:** 1 Department of Public Health, College of Health Sciences, Mettu University, Mettu, Ethiopia; 2 Department of Midwifery, College of Health Sciences, Dire Dawa University, Dire Dawa, Ethiopia

**Keywords:** Long act reversible contraceptive, Family planning methods, HIV, HIV positive women, Mettu

## Abstract

**Background:**

Identifying the determinants of long-acting contraceptive utilization and managing the sexual and reproductive health of HIV-infected women is critical to reducing HIV transmission and maternal mortality. However, the determinants of long-acting contraceptive utilization have not been well understood in resource-limited settings like Ethiopia. The aim of this study was to identify determinants of long-acting reversible contraceptive utilization among HIV-positive women on ART in southwest Ethiopia.

**Methods:**

A facility-based, unmatched case control study was conducted from July 24 to August 28, 2021, in south-west Ethiopia. The study participants were HIV positive women, with a sample size of 109 cases and controls. An interviewer administered a questionnaire, and a check list was used for data collection. A systemic random sampling technique was used to collect data from cases and controls. Bivariate and multivariable logistic regressions were employed to determine the determinants of LARC utilization among HIV-positive women. To demonstrate the strength of the determinant, the odds ratio was calculated with 95% confidence intervals, and a *P*-value less than 0.05 was used to declare statistical significance.

**Result:**

A total of 324 women (108 cases and 216 controls) of reproductive age who were HIV positive were interviewed, with a response rate of 99.0%. urban residence (AOR = 2.67, 95%CI: 1.23- 5.77), having formal education (AOR = 2.93, 95% CI:1.36, 6.34), being counseled by health care provider (AOR = 5.42,95% CI: 2.67–11.03), no future fertility intention (AOR = 2.87, 95% CI:1.44–5.70), having CD4 count less than 500 cell/mm^3^ (AOR = 4.18,95% CI:2.12–8.23), having information of HIV transmission from mother to child (AOR = 3.65,95% CI:1.49–8.95),not using condom during sexual intercourse (AOR = 4.86,95% CI:2.46–9.62),,having knowledge towards LARC (AOR = 2.38,95% CI:1.24–4.58) and attitude towards LARC (AOR = 6.41,95%CI:3.16–13.0) were independent determinants of LARC utilization among HIV positive women.

**Conclusion and recommendation:**

Women being counseled by a health care provider, having no future fertility intention, and having a CD4 count less than 500 cells/mm^3^ were found to be determinants of long-acting contraceptive method utilization among HIV-positive reproductive-age women. Also, our study supports the WHO Strategic Concepts for Improving the Links between Family Planning and HIV/AIDS Policy, Programs, and Services. It is recommended that Health care providers should use these factors as base line during family planning counseling and service delivery.

## Introduction

Contraception is defined as the ability of individuals and couples to anticipate and reach their desired number of children through the use of contraceptive methods [1]. Contraceptive use is an important intervention for improving maternal and child health, preventing HIV infections, and improving the overall well-being of entire families through the prevention of unintended pregnancy and unsafe abortion [1–3]. Preventing unwanted pregnancy among HIV-positive women is one of the best strategies to prevent new HIV infections in newborn children. Among family planning methods that prevent unwanted pregnancies, long-acting reversible contraceptives are important for reproductive-age women infected with HIV [2–4].

Long-acting reversible contraceptives work more effectively than short-term contraceptives and are reversible in case a wanted or planned child is needed in the couple [2, 5]. Long-acting reversible contraceptives are hormonal contraceptives, which consist of either estrogen or progesterone, like the Implants and Jedelle categories of contraceptive methods, and free of hormones like the Intra uterine contraceptive device (IUCD) modern contraceptive subclasses of contraceptive methods [1, 5]. Dual protection, which includes use of a reliable hormonal contraceptive method like injectable, implanon, jedelle, and IUCD, and a barrier method like using a male or female condom, is encouraged to prevent further transmission of HIV from those who practiced multisexual partners [6].

In sub-Saharan countries, unintended pregnancy among women living with HIV ranges from 53 percent to 84% [7], and the magnitude of unintended pregnancy in Ethiopia was 27.1% [8].Women with HIV infection, like other women, may wish to plan their pregnancy, limit their family, or avoid pregnancy [1].

The Integration HIV and FP services and effective counseling were very curtail methods of update long act reversible contraceptive uptake [9].

The contraceptive prevalence rate has steadily increased from 8% in 2000 to 41.4, and the unmet need for family planning methods has declined from 37% in 2000 to 22% in 2016, according to an updated Ethiopian demographic health survey. The most popular contraceptive methods used were injectable (27%), implants (9%), and IUCD (2%) [10].

According to a WHO fact sheet, reversible long-acting contraceptives are important for HIV-positive women because they effectively prevent unintended pregnancy, and due to this, the following problems are reduced: mother-to-child transmission, the number of new HIV infections among children, and the number of HIV/AIDS-related maternal deaths [7].

According to different studies done in Ethiopia, LARCs utilization has been very low among HIV-positive reproductive-age women [2, 11]. To lower HIV transmission and maternal mortality in areas with a high HIV prevalence, it is essential to manage the sexual and reproductive health of HIV-infected women. Additionally, despite the study area being in high-magnitude areas for HIV/AIDS risk priorities [12], no study has been attempted concerning long-acting reversible contraceptive utilization in the specified study area.

However, the determinants of long-acting reversible contraceptive utilization have not been well understood in resource-limited settings like Ethiopia. Understanding the determinants is beneficial in a way that patients as well as caretakers can intervene on those determinants. The study will contribute to filling the existing information gap and hence suggest proper intervention measures for LARC utilization, which in turn will reduce unintended pregnancy among HIV-positive women and infant HIV infection. Thus, this study was aimed at identifying the determinants of long-acting reversible contraceptive utilization among HIV-positive reproductive-age women attending care in ART clinics in Mettu Karl Specialized Hospital, Southwest Ethiopia.

## Methods and materials

### Study setting and design

An institutionally based, unmatched case–control study was conducted from July 24 to August 28, 2021, at Mettu Karl specialized hospital in Mettu town, which is found in Oromia regional state, south-west Ethiopia. Mettu town is located about 600 km from Addis Ababa, the capital city of Ethiopia. The Mettu Karl Specialized Hospital gives different inpatient and outpatient services to the population in the surrounding Illu Aba Bor zone and comprehensive service to south western Ethiopia with a catchment population of more than 2.4 million. The hospital has been providing ART services for free since 2006 G.C. Currently, there are 1607 patients, both male and female, on ART at Mettu Karl Specialized Hospital. Among these 900 are reproductive-age women.

### Source population and study population

All HIV-positive reproductive age women (15–49 years) who had ART follow-up in MKSH were the source population, and those women who fulfilled the inclusion criteria during the data collection period were the study population.

All HIV-positive women of reproductive age women (15–49) who were using long-acting contraceptive methods were considered cases, while all HIV-positive women of reproductive age who were not using contraceptive methods were considered controls.

All HIV-positive reproductive-age women (15–49) who use short-acting contraceptive methods and women who were severely ill during the data collection period were excluded from the study.

### Sample size determination and sampling technique

The sample size was determined based on the double population proportion formula using the StatCalc function in Epi Info version 7. By considering 95% CI, 80% power, a case-to-control ratio of 1:2, and taking different sample sizes, different risk factors for long-acting contraceptive utilization among HIV-positive women were identified. The maximum sample size was obtained, and accordingly, when deciding about family planning methods, the odds of long-acting contraceptive utilization were higher among women who decided about contraceptive use jointly with their partner than those who used to decide by themselves, according to a previous study conducted in Bahir Dar [13]. While the proportion of exposure among controls was found to be 31.3%, the percent of cases with exposure was 16.1%, and the odds ratio was 0.42. Accordingly, this yields a maximum sample size of 103 cases and 206 controls. By adding a 5 percent non-response rate, the final sample size required for the study was 109 cases and 218 controls, and the total calculated sample size was 327. Using systematic random sampling techniques, the required sample size was included in the study.

### Data collection tool, method and procedure

Data was obtained using a standardized questionnaire to interview all eligible women and a data extraction program that was adapted from a prior study [2, 11, 14, 15]. The questionnaire mainly addressed socio-demographic variables of mothers (age, residence, marital status, educational level of women, educational level of husbands, and occupation), reproductive health variables (parity, number of children, contraceptive use before HIV positivity, counseling on LACMs by providers, future fertility intention, and decision of FP methods), and HIV and sexual relations variables (duration on ART, STI history, use of a condom during sexual intercourse, and having a multisexual partner). A checklist was used for medical history variables (clinical WHO staging, disclosure of HIV status, CD4 count, time of HIV diagnosis, and ART user). Data collectors were two clinical nurses and one public health officer supervisor, after a two-day training and orientation period. Every day, the primary investigator examined the data for completeness.

### Measurement

#### Knowledge about LARC

Knowledge was measured among those who reported they had heard about LARC methods. The total knowledge question had five items, and after computing all variables, the minimum score was zero and the maximum was five. To measure the knowledge, it was categorized based on the mean of knowledge as: "Good knowledge"- above mean and "Poor knowledge" exactly mean and below [15].

#### Attitude toward LARC

Attitude was measured among those who reported having beliefs about LARC methods. The total number of questions about attitude toward LARC was four, and after computing all variables, the minimum score was zero and the maximum was eight. To measure the attitudes of participants towards LARC, responses were categorized based on the mean of attitude responses as: "favorable attitude"- above mean and "unfavorable attitude" exactly mean and below [15].

### Data quality control

Data quality was ensured by providing training for data collectors and strict supervision during data collection. A pretest was conducted on 5% of the sample size in the Bedelle General Hospital before the actual data collection.

### Data processing and analysis

The data were manually checked for completeness, coded, and cleaned before being entered into a computer. Then, it was entered into Epi Data version 3.1 and exported into SPSS version 20 for analysis. Data exploration was conducted to assess the completeness, and descriptive statistics like frequencies, tables, and figures were used to describe background variables. A bivariable logistic regression analysis was done for each independent variable, and then, those variables with *p* values ≤ 0.25 were entered into a multivariable logistic regression to control possible confounders. The backward stepwise logistic regression variable selection method with a *P*-value less than 0.05 and an AOR with their respective 95% CI was used to identify independent predictors for long-acting reversible contraceptive utilization. The model’s fitness was tested using the Hosmer and Lamshow goodness of fit test, and the model was declared fit (*P* = 0.250). Finally, the results were presented using tables, graphs, and narration.

## Results

### Socio-demographic characteristics

A total of 324 women (108 cases and 216 controls) of reproductive age on antiretroviral therapy follow-up were included in the study, with a response rate of 99 percent. The mean age with corresponding standard deviation for cases and controls were 33.04 + 7.87 SD and 31.42 + 7.00 SD, respectively. Regarding the age distribution of study participants, fifty-six (27.5%) cases and one hundred forty-eight (72.5%) controls were in the 20–34 age range (Table 1).

**Table 1:** Socio-demographic characteristics of HIV positive women attending ART in Mettu Karl comprehensive specialized hospital, 2021

Variable	Category	Cases (*N* = 108)	Controls (*N* = 216)
**Age**	≤ 20 years	4(3.7%)	14(6.5%)
	20–34 years	56(51.9%)	148(68.5%)
	≥ 35 years	48(44.4%)	54(25.0%)
**Residence**	Urban	92(85.2%)	145(67.1%)
	Rural	16(14.8%)	71(32.9%)
**Marital status**	Married/live together	88(81.5%)	167(77.3%)
	single	12(11.1%)	39(18.1%)
	Others	8(7.4%)	10(4.6%)
**Religion**	Orthodox	49(45.4%)	124(57.4%)
	Muslim	18(16.7%)	32(14.8%)
	Protestant	38(35.2%)	57(26.4%)
	Others	3(2.8%)	3(1.4%)
**Ethnicity**	Oromo	74(68.5%)	142(65.7%)
	Amhara	22(20.4%)	53(24.5%)
	Gurage	9(8.3%)	13(6.0%)
	Others	3(2.8%)	8(3.7%)
**Mothers’ Educational level**	No formal education	18(16.7%)	66(30.6%)
	Formal education	90(83.3%)	150(69.4%)
**Occupation**	House wife	48(44.4%)	94(43.5%)
	Gove’s	20(18.5%)	51(23.6%)
	Self and private business	32(29.6%)	60(27.8%)
	others	8(7.4%)	11(5.1%)
**Husbands’ educational level (*n* = 281)**	No formal education	73(73.0%)	119(65.7%)
	Formal education	27(27.0%)	62(34.3%)

### Reproductive health related variables of LARC utilization

Among the respondents, 98 (90.7%) of cases and 151 (69.9%) of controls had ever given birth. Of total participants, 96 (88.9%) of cases and 129 (59.7%) of controls get pregnant before HIV positivity, and for both groups, more than half of women had ever used contraception before becoming HIV positive (Table 2).

**Table 2:** Reproductive health characteristics of HIV positive women at attending ART in Mettu Karl comprehensive specialized hospital, 2021

Variables	Category	Cases (*N* = 108)	Controls (*N* = 216)
**Ever give birth**	Yes	98(90.7%)	151(69.9%)
	No	10(9.3%)	65(30.1%)
**Number of children**	One	33(33.7%)	49(32.5%)
	Two	20(20.4%)	64(42.4%)
	Three	22(22.4%)	30(19.9%)
	Four and above	23(23.5%)	8(5.3%)
**Ever pregnant before HIV + **	Yes	96(88.9%)	129(59.7%)
	No	12(11.1%)	87(40.3%)
**Ever used contraceptive before HIV + **	Yes	84(77.8%)	133(61.6%)
	No	24(22.2%)	83(38.4%)
**History of contraceptive use after HIV + **	Yes	86(79.6%)	120(55.6%)
	No	22(20.4%)	96(44.4%)
**Counselled on LARC methods by health care provider**	Yes	84(77.8%)	101(46.8%)
	No	24(22.2%)	115(53.2%)
**Future fertility intention**	Yes	42(38.9%)	135(62.5%)
	No	66(61.1%)	81 (37.5%)
**Decision of contraceptive use**	Wife only	55(50.9%)	95(44.0%)
	Jointly	43(39.8%)	81(37.5%)
	Partners only	10(9.3%)	40(18.5%)

### HIV and sexual related determinants of LARC utilization

This study indicates that of the total participants, 58 (53.7%) cases and 118 (54.6%) controls had been diagnosed with HIV within the last 8 years. Among the respondent’s 28 (26.0%) cases and 126 (58.3%) controls had used a condom during sexual intercourse (Table 3).

**Table 3:** HIV and sexual related Characteristics of HIV positive reproductive age women attending ART in Mettu Karl comprehensive specialized hospital, 2021

Variables	Category	Cases (*N* = 108)	Controls (*N* = 216)
**Duration on ART**	< 8 years	58(53.7%)	118(54.6%)
	> / = 8 years	50(46.3%)	98(45.4%)
**Recent CD4 count**	< 500cell/mm^2^	67(62.0%)	64(29.6%)
	≥ 500cell/mm^2^	41(38.0%)	152(70.4%)
**Have information of HIV transmitted to child**	Yes	94(87.0%)	161(74.5%)
	No	14(13.0%)	55(25.5%)
**Use condom during sexual intercourse**	Yes	28(26.0%)	126(58.3%)
	No	80(74.0%)	90(41.7%)
**Practice multi sexual partner**	Yes	12(11.1%)	16(7.4%)
	No	96(88.9%)	200(92.6%)

### WHO clinical stage of respondents

**Fig. 1:**
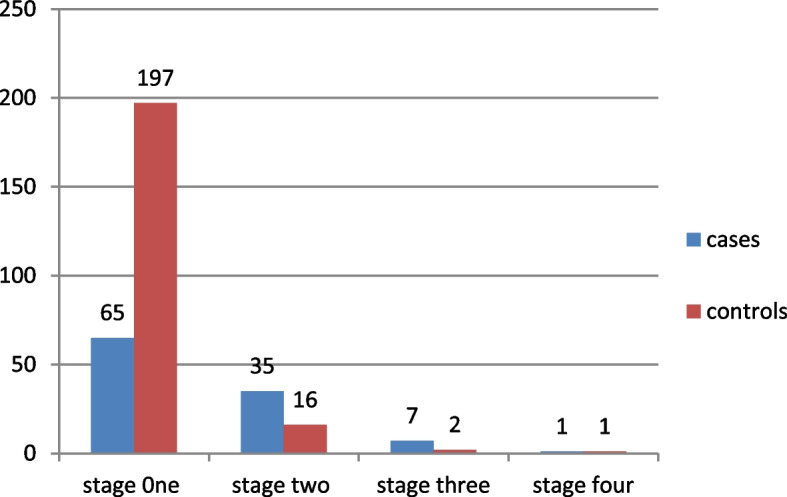
WHO clinical stage among HIV reproductive age women on ART in MKSH, South West Ethiopia, 2021

Out of all HIV-positive women of reproductive age who attend an ART clinic, 65 (60.2%) are cases and 197 (91.2%) are controls (Fig. 1).

### Knowledge and attitude of respondents towards LARC utilization

In this study, knowledge and attitude of participants were categorized after computing the mean and standard deviation of the respondent. As a result, the mean of knowledge was 7.99 ± 1.02 and the mean of attitude was 7.06 ± 2.06 (Table 4).

**Table 4:** Knowledge and attitude of HIV positive reproductive age women attending ART in Mettu Karl comprehensive specialized hospital, 2021

Variable	Category	Case (*N* = 108)	Control (*N* = 216)
**Knowledge**	Good Knowledge	75(69.4%)	97(45.0%)
	Poor Knowledge	33(30.6%)	119(55.0%)
**Attitude**	Favorable	71(65.7%)	74(34.3%)
	Unfavorable	37(34.3%)	142(65.7%)

### Determinant factors of long acting contraceptive utilization among HIV positive reproductive age women

Several factors, such as socio-demographic, reproductive health, method-related, HIV-related, and sexual-related factors, were tested for the presence of an association with LARC utilization using bivariable regression analysis.

In multivariable logistic regression, variables like being an urban resident, having formal education, being counseled on LARC by health professionals, having no future fertility intentions, having CD4 counts of less than 500 cells/mm^3^, having information about HIV transmission from mother to child, not using a condom during sexual intercourse, and having good knowledge of and a favorable attitude toward LARC methods were associated with LARC utilization. The odds of long-acting reversible contraceptive utilization were 2.67 times higher among urban residents as compared to rural residents (AOR = 2.67, 95CI (1.23, 5.77)). The odds of long-acting reversible contraceptive utilization were 2.93 times higher among women who had formal education as compared to those who didn’t have formal education (AOR = 2.93, 95%CI: 1.36, 6.34). Women who had received LARC counseling were 5.42 times more likely to use LARC (AOR = 5.42, 95% CI: 2.67–11.03) than those who had not received counseling. Women who had CD4 less than 500 cells/mm^3^ were 4.18 times more likely to utilize LARC than women who had CD4 greater than 500 cells/mm^3^ (AOR = 4.18,95%CI:2.12,8.23).Women who knew HIV is transmitted from mother to child were 3.65 times more likely (AOR = 3.65, 95% CI: 1.49–8.95) to utilize LARC than those who didn’t know. Women who used a condom during sexual intercourse were 4.86 times more likely (AOR = 4.86; 95% CI: 2.46–9.62) to utilize LARC than those who didn’t. The odds of LARC utilization were about 2.87 among women who were not intended to give birth in the future compared to those who were intended to give birth (AOR = 2.87, 95%CI: 1.44–5.70).The odds of LARC utilization were about 2.38 times higher among women who had good knowledge of LARC methods than among those who had poor knowledge (AOR = 2.38, 95% CI: 1.24–4.58). The odds of LARC utilization were about 6.41 times higher among women who had a favorable attitude toward LARC methods than those who had an unfavorable attitude (AOR = 6.41, 95% CI: 3.16–13.0) (Table 5).

**Table 5:** Bivariate and multivariate analysis of determinants of LARC utilization among HIV positive women attending ART Clinic in MKSH in, South West Ethiopia, 2021

Variables	Category	Cases *N* = 108	Controls *N* = 216	COR 95%CI	AOR 95%CI	*p*-value
**Age**	≤ 20 years	4(22.2%)	14(77.8%)	1.0	1.0	
	20–34 years	56(27.5%)	148(72.5%)	1.32(0.41,4.19)	0.49(0.09,2.78)	0.42
	≥ 35 years	48(47.1%)	54(52.9%)	3.11(0.96,10.09)	0.84(0.13,5.18)	0.84
**Place of Residence**	Urban	92(38.8%)	145(61.2%)	2.81(1.54,5.14)	2.67(1.23, 5.77)	0.012*
	Rural	16(18.4%)	71(81.6%)	1	1	
**Marital Status**	Married	88(34.5%)	167(65.5%)	1.0	1.0	
	single	12(23.5%)	39(76.5%)	0.58(0.29,1.17)	2.23(0.61,8.11)	0.22
	Others	8(44.4%)	10(55.6%)	1.52(0.58,3.99)	0.56(0.13,2.36)	0.42
**Mothers Educational Level**	No formal education	18(21.4%)	66(78.6%)	1.0	1.0	
	Formal education	90(37.5%)	150(62.5%)	2.2(1.23,3.94)	2.93(1.36,6.34)	0.006*
**Have Children**	Yes	98(39.4%)	151(60.6%)	4.22(2.07,8.61)	2.33(0.93,5.89)	0.07
	No	10(13.3%)	65(86.7%)	1.0	1.0	
**Counselled on LARC by Health professional**	Yes	89(49.7%)	90(50.3%)	3.99(2.35,6.75)	5.42(2.67,11.03)	0.000*
	No	19(13.1%)	126(86.9%)	1.0	1.0	
**Future Fertility intension**	Yes	42(23.7%)	135(76.3%)	1.0	1.0	
	No	66(44.9%)	81(55.1%)	2.62(1.63,4.21)	2.87(1.44,5.70)	0.003*
**CD4 in cells/mm3**	< 500cell/mm^2^	67(51.1%)	64(48.9%)	3.88(2.39,6.31)	4.18(2.12,8.23)	0.000*
	> / = 500cell/mm^2^	41(21.2%)	152(78.8%)	1.0	1.0	
**HIV transmission from mother to child**	Yes	94(36.9%)	161(63.1%)	2.29(1.21,4.35)	3.65(1.49,8.95)	0.005*
	No	14(20.3%)	55(79.7%)	1.0	1.0	
**Use condom during sexual intercourse**	Yes	28(18.2%)	126(81.8%)	1.0	1.0	
	No	80(47.%1)	90(52.9%)	4.00(2.40,6.65)	4.86(2.46,9.62)	0.000*
**Knowledge**	Good	75(43.6%)	97(56.4%)	2.79(1.71,4.55)	2.38(1.24,4.58)	0.009*
	Poor	33(21.7%)	119(78.3%)	1.0	1.0	
**Attitude**	Favourable	71(49.0%)	74(51.0%)	3.67(2.26,5.99)	6.41(3.16,13.00)	0.000*
	Unfavourable	37(20.7%)	142(79.3%)	1.0		

## Discussion

This study’s findings revealed that women who resided in urban, had formal education, were counseled on LARC by health professionals, had no future fertility intentions, CD4 < 500 cell/mm3, had information about HIV transmission from mother to child, did not use a condom during sexual intercourse, and had good knowledge of and a favorable attitude toward LARC methods were associated with LARC utilization.

This study has found that the place of residence of the women is significantly associated with the utilization of LARC. Women who resided in urban were 2.6 times more likely to use LARC as compared to rural residents. This finding is supported by studies done in Ethiopia [16] and the Borana district, northeast Ethiopia [6]. The reason for study comparability might be due to urban residents being easily accessible to the contraceptive delivery service.

In this study’s findings, women with formal education were almost three times more likely to use LARC as compared to those who had not attended formal education. This finding was consistent with studies done in Borena district, Adaba town, Arsi zone, Oromia, Bahirdar, Ethiopia, and Malawi [6, 13, 17, 18]. This could be due to the fact that educated people tend to adopt modern values, have the opportunity and better access to adequate information about contraceptives, and have greater autonomy to make decisions and use long-acting contraceptives.

The result of this study also showed that women who had been counseled about LARC by a health care provider were statistically associated with LARC utilization as compared with women who had not been counseled. This result is supported by a study conducted in Bahir Dar Town, Northern Ethiopia [13], and Dangila Town, Hawi Zone, Ethiopia [19], which indicated that getting information or counseling about family planning from a health care provider was significantly associated with the use of long-acting reversible contraceptives. The possible reason for the similarity of the findings might be because the health care provider delivered similar information regarding LARC service utilization to their customers.

Therefore, counseling on LARC by health care workers was very important to increase LARC uptake and prevent unintended pregnancy among HIV-positive women through integration of the service with other departments besides the family planning unit, like the ART center and other chronic disease departments.

In this study, women who had no future fertility intention had a 2.87 times higher demand for long-acting reversible contraceptive methods than those who had a future fertility intention. This was similar to the study done in Ambo, Northwest Ethiopia, and Arbaminch, Ethiopia [2, 15]. This may be due to the fact that women who have no future fertility intentions prefer long-acting contraceptive methods, which are more effective than short-term hormonal contraceptives and barrier methods.

Being informed of HIV transmission from mother to child is one of the factors that had a significant association with LARC utilization. In this study, women who had information about HIV transmission from mother to child were 3.65 times more likely to utilize LARC than those who didn’t have that information. The possible reason they demand to use LARC might be due to their fear of HIV transmission to their offspring.

Similarly, this study revealed that CD4 level is significantly associated with LARC utilization. The odds of having CD4 less than 500 cells/mm^3^ were at most four times more likely to utilize LARC as compared to those who had CD4 >  = 500 cells/mm^3^. The variation might be in women who had a lower CD4 count and were exposed to frequent counseling, which promotes the utilization of LARC to prevent HIV transmission to the child.

In terms of condom use during sexual intercourse, women who did not use a condom during sexual intercourse were 4.86 times more likely to use LARC than those who did. The explanation for this could be a misunderstanding that using a condom alone as contraception is another important factor that significantly affects LARC utilization, along with knowledge of LARC methods. The study revealed that women who had good knowledge about LARC were twice as likely to use it as those who had poor knowledge. This result was supported by a study done in Ambo Town [15], Adaba Town [18] and Bahir Dar health facilities in Northwest Ethiopia [20]. The possible reason might be due to methodological and socio-demographic similarities.

In this study, HIV-positive women of reproductive age who had a favorable attitude toward LARC were 6.4 times more likely to use LARC compared to those who had an unfavorable attitude. This finding was comparable to that of a study conducted in the East Gojjam Zone of Northwest Ethiopia [21], Ambo Town [15] Mekele town [22], Northern Ethiopia [23] and Bari Dar town [20]. This comparability of findings could be due to the fact that women who had a favorable attitude had self-confidence or self-motivation and no method-related confusion or fear of using LARC methods.

## Limitations

The study was prone to social desirability bias because it depended solely on women’s responses while excluding male partner engagement. It also relied on participants self-reported data. However, a supervisor was present to ensure that such biases were minimized and that any potential ambiguities or misunderstandings were clarified, as well as to protect participants’ privacy during the interview.

## Conclusion

In one area of RH, family planning (FP), connections to HIV interventions are crucial. The possibility to expand access to contraception among HIV service recipients who do not want to get pregnant or to ensure a safe and healthy pregnancy and delivery for those who do so is provided by integrating FP services into HIV prevention, treatment, and care programs [24].

In this study, we found that urban residence, having formal education, being counseled by a health care provider, having no future fertility intention, having a CD4 count less than 500 cells/mm3, having information about HIV transmission from mother to child, not using a condom during sexual intercourse, and having knowledge of and an attitude towards LARC were independent factors significantly associated with long-acting reversible contraceptive utilization. Health care providers should give effective counseling for HIV-positive women about childbearing and pregnancy issues, in addition to LARC utilization, using a condom during sexual intercourse to suppress viral load, and advancing the knowledge and attitude of mothers about long-acting contraceptive methods and Further studies are needed on the association of LARC utilization among HIV-positive women with their quality of life.

### Data sharing statement

The datasets used and/or analyzed during the current study are available from the corresponding author on reasonable request.

## Data Availability

Data will be available from the corresponding author upon request.

## Abbreviations

AOR: Adjusted odds ration
ART: Anti retro therapy
CD4: Cluster of differentiation 4
FP: Family Planning
HIV: Human immunology virus
IUCD: Intra-uterine contraceptive device
LACU: Long act contraceptive utilization
LARC: Long act reversible contraceptive
MCH: Maternal and child health
MKSH: Mettu Karl specialized hospital
PMTCT: Prevention of mother to child
WHO: World health organization

## References

[1] Health FDR of EM of National Edition T, Ababa A. National Guideline for Family Planning Services In Ethiopia Third Edition. 2020:1–77.

[2] Yirsaw B, Gebremeskel F, Gebremichael G, Shitemaw T. Determinants of long acting contraceptive utilization among HIV positive reproductive age women attending care at ART clinics of public health facilities in Arba Minch town, Southern Ethiopia, 2019 : a case control study. AIDS Res Ther. 2020;8:1–8. 10.1186/s12981-020-00288-x PMC7296955 32539743

[3] Davies NECG, Ashford G, Bekker L-G, Chandiwana N, Cooper D, Dyer SJ (2018). Guidelines to support HIV-affected individuals and couples to achieve pregnancy safely: update 2018. South Afr J HIV Med.

[4] McCoy SI, Buzdugan R, Ralph LJ, Mushavi A, Mahomva A, Hakobyan A (2014). Unmet need for family planning, contraceptive failure, and unintended pregnancy among HIV-infected and HIV-uninfected women in Zimbabwe. PLoS One.

[5] EngenderHealth (2014). Integrating family planning and antiretroviral therapy: a client-oriented service model.

[6] Care P (2021). Dual Contraceptive use and associated factors among reproductive age group on antiretroviral therapy in Borena District, Northeast Ethiopia : a cross-sectional study.

[7] Plan G (2011). Preventing unintended pregnancies and HIV.

[8] Habte D, Teklu S, Melese T, Magafu MGMD (2013). Correlates of unintended pregnancy in Ethiopia: results from a national survey. PLoS ONE.

[9] Family Health International (2010). Study of family planning and HIV integrated services in five.

[10] Central Stastical Agency (CSA), and ICF, Addis Ababa, Ethiopia and Rockvillle, maryland U. Ethio[ian health demographic and health survey. 2016.

[11] Tewabe T, Ayalew T, Abdanur A, Jenbere D (2020). Heliyon Contraceptive use and associated factors among sexually active reproductive age HIV positive women attending ART clinic at Felege Hiwot Referral Hospital, Northwest Ethiopia: a cross-sectional study. Heliyon.

[12] Office C (2020). HIV Prevention in Ethiopia National Road Map.

[13] Kebede HG, Nahusenay H, Birhane Y, Tesfaye DJ (2015). Assessment of contraceptive use and associated factors among HIV Positive Women in Bahir-Dar Town. Northwest Ethiopia.

[14] Demeke CA, Kasahun AE, Yimenu DK (2020). Utilization pattern of long-acting and permanent family planning methods and associated factors : a community-based cross-sectional study in.

[15] Soboka DR (2016). Determinants of long-acting reversible contraceptive methods utilization among married women of reproductive age group in Ambo Town, Oromia Region, West Ethiopia, 2016 : a case control study.

[16] Dasa TT, Kassie TW, Roba AA, Wakwoya EB, Kelel HU (2019). Factors associated with long-acting family planning service utilization in Ethiopia: a systematic review and meta-analysis. Contracept Reprod Med.

[17] Habte D, Namasasu J (2015). Family planning use among women living with HIV: knowing HIV positive status helps - results from a national survey. Reprod Health..

[18] Fekadu H (2017). Prevalence and determinant factors of long acting contraceptive utilization among married women of reproductive age in Adaba Town, West Arsi Zone. J Womens Health Care.

[19] Genet E, Abeje G, Ejigu T (2015). Determinants of unmet need for family planning among currently married women in Dangila town administration, Awi Zone, Amhara regional state; a cross sectional study. Reprod Health.

[20] Tesfa E, Gedamu H (2018). Factors associated with utilization of long term family planning methods among women of reproductive age. BMC Res Notes.

[21] BewketZeleke L, Gella MM, AlmawDerseh H, Alemu AA, AbebeKassahun E, Gelaw KA (2019). Utilization of long-acting contraceptive methods and associated factors among female health care providers in East Gojjam Zone, Northwest Ethiopia, in 2018. Biomed Res Int.

[22] Alemayehu M, Belachew T, Tilahun T (2012). Factors associated with utilization of long acting and permanent contraceptive methods among married women of reproductive age in Mekelle town, Tigray region, north Ethiopia. BMC Pregnancy Childbirth.

[23] Yalew SA, Zeleke BM, Teferra AS (2015). Demand for long acting contraceptive methods and associated factors among family planning service users, Northwest Ethiopia: a health facility based cross sectional study. BMC Res Notes.

[24] Planning F. Strategic considerations for strengthening the linkages between family planning and HIV / AIDS policies, programs, and services.

